# A systematic mapping review of factors associated with willingness to work under emergency condition

**DOI:** 10.1186/s12960-021-00622-y

**Published:** 2021-06-24

**Authors:** Hamideh Nafar, Emir Tahmazi Aghdam, Naser Derakhshani, Nadia Sani’ee, Sakineh Sharifian, Salime Goharinezhad

**Affiliations:** 1 Department of Health Services Management, School of Health Management and Information Sciences, Iran University of Medical Sciences, Tehran, Iran; 2 Health Management and Economics Research Center, Health Management Research Institute, Iran University of Medical Sciences, Tehran, Iran; 3 Department of Medical Librarianship and Information Sciences, School of Health Management and Information Sciences, Iran University of Medical Sciences, Tehran, Iran; 4 Nursing Care Research Center, Iran University of Medical Sciences, Tehran, Iran; 5 Department of Nursing Management, School of Nursing and Midwifery, Iran University of Medical Sciences, Tehran, Iran; 6 Preventive Medicine and Public Health Research Center, Psychosocial Health Research Institute, Iran University of Medical Sciences, P.O. Box: 1449614535, Tehran, Iran

**Keywords:** Willingness to work, Retention, Emergency condition, Health workforce, Mapping review

## Abstract

**Introduction:**

An effective response to an emergency situation relies on health care workers’ preparedness. The main purpose of this study was to provide a comprehensive overview of relevant studies regarding the willingness to work in emergency and disaster situations, describe and classify the most important challenges and solutions, identifying knowledge gaps in the literature which could inform future research.

**Methods:**

In this Systematic Mapping Review required information was searched from PubMed, Scopus, the web of science, Embase databases, and Google scholar search engine in the period 2000–2020. Data were analyzed using a content framework analysis.

**Results:**

From 2902 article search results, 26 articles met the inclusion criteria. The studies varied in terms of aim, study design, and detail of reporting. The results showed that nearly three-quarters of studies were conducted in high and middle-income countries. Most of the studies were published in 2020 due to the COVID-19 pandemic. Also, the most common types of crises reported in the included studies were emerging and re-emerging infectious diseases. The results show that most of the problems were in the dimension of mental and psychological issues, personnel health concerns, and management relationship with personnel.

**Conclusion:**

This mapping review illustrated a big picture of health workers' resilience in disaster conditions. This review presents an overview of different kinds of strategies that address the challenges.

One of the most important challenges in health workforce retention is poor communication between managers and staff. Being away from family, which leads to mental fatigue, puts staff in moral dilemmas. Attracting adequate health professionals, especially volunteers and regulating the shifts of health personnel in crisis time will largely prevent burnout.

**Supplementary Information:**

The online version contains supplementary material available at 10.1186/s12960-021-00622-y.

## Introduction

Human resource is one of the main elements that affect the efficiency of systems and has a key role in healthcare systems when comparing to other elements. This is why it has undeniable importance among all the resources used in providing hospital services. Without the trained and qualified human resources, the hospitals’ affairs will be disrupted [1]. Deficiencies, and reducing the efficiency and motivation of the human resources will diminish the quality of patient care services. One of the common problems of hospitals is to determine their needs of the human resources, and because of that, the management of the human resources should be an important part of the healthcare system’s planning [2].

There is room for debate in the definition of “the emergency situation” that has been widely used as an academic concept [1]. In general, the change of situation from usual to unusual is dangerous and can cause a crisis [3, 4]. Crises are rare that occur by unpredictable factors [5]. When a natural or man-made emergency goes beyond the capability and capacity of a society or system, it is called a disaster. If the disaster occurs, all aspects of human life will be affected and the consequences will appear in the form of death, physical injuries, damage to assets and infrastructures, and the imposition of social and economic costs.

Disasters have always threatened human life. According to conducted researches, the number of disasters is increasing; for instance, the number of worldwide disasters in the year of 2019 was 409. Each type of disaster is concomitant with its own emergencies that directly and indirectly affect the healthcare system [3]. If the critical situation continues, the government and the crisis committees of healthcare centers should determine where patients will be treated and who should provide services [6].

In an emergency situation, healthcare centers and hospitals have to provide services for a large number of patients and the continuity of these services requires meticulous planning of healthcare officials and policy-makers of the countries. At the time of emergency and disaster, the most important components of planning are as follows: enhancing the motivation of personnel to attend their job and encouraging them to provide effective services. These components can prevent problems like increasing the time and workload of health workers. In addition to the fact that the health workers are required to perform their duties effectively and accurately, they are also exposed to the risk of infection, burnout, separation from family, and emotional pain caused by the death of patients and colleagues.

The main variables that affect the absence of personnel at the time of disaster are as follows: (1) the personal willingness of the staff to accept the risk of performing duties. (2) Their willingness to show their ability at work. According to the report from hospitals and public health centers, a substantial percentage of personnel’s absence occurs during incidents like pandemics and terrorist cases such as chemical, biological and nuclear incidents [4, 6]. Also, some literature indicated that after natural disasters absenteeism and job satisfaction significantly have decreased [7].

Some studies emphasized that a purposeful plan that supports patients, personnel’s families, and substructures of hospitals can enhance the personnel’s willingness to work [8]. Planning about increasing the capacity of hospitals that consists of providing sufficient space, equipment, and human resources during incidents and disasters is one of the basic measures of service delivery systems before the occurrence of an emergency. In these circumstances, it is necessary to increase the capacity and the number of human resources in a coordinated manner in healthcare systems [9, 10].

The absence of personnel in hospital disaster and emergencies is an issue that has not been discussed sufficiently [11]. Researchers by considering the high rate of absenteeism among nurses in the country (about 20 percent) believe that having purposeful plans to protect patients, personnel’s families, and hospitals’ substructures can improve the personnel’s willingness to work [12].

Studies have shown that the main problem of Iran’s hospitals is the paucity and inappropriate distribution of human resources. These issues become serious in the time of disaster and urgency. Considering the vital role of human resources in the quality and quantity of provided services in hospitals and the lack of research in this field, it is necessary to conduct more studies about this subject.

Furthermore, according to studies, the management of human resources in the condition of emergency and disaster should be considered in emergency planning. It is important for policy-makers and healthcare managers to know the main factor associated to ability and willingness to care especially in disaster situation and prepare health care systems to increase their resilience in disaster. The objective of this research is to examine the strategies of enhancing the willingness to work among the health workforce and reduce their absence in the time of emergency or disaster.

## Methods

A systematic mapping review was conducted to explore research into willingness to work under disaster condition and associated interventions to maintain the health workers. Mapping reviews do not assess the quality of studies, but rather define and categorize the existing evidence base. This map review focuses on two main themes. First, it explores studies that have been developed for interventional strategies. Second, it summarizes the research gaps that require to be addressed in future studies.

### Sources of information

The required information was searched in PubMed, Scopus, Web of science, Embase and Google Scholar search engines, without a time limit and in English. The search strategy was developed under a medical librarian supervision and consultation (NS).

The keywords used for searching the studies were: “Healthcare Worker”, “Healthcare Personnel”, “Health Manpower”, “Healthcare Provider”, “Allied Health Personnel”, “Medical Staff”, personnel, health, Maintenance, retention, sustain, “Personnel Turnover,” “Employee Turnover”, disaster, Emergencies, pandemic. Also, a search in the subject area from specialized journals, the World Health Organization website and a review of the final list of articles for inclusion in the study was done manually (Table 1).

**Table 1:** Complete search strategy for all databases

Database	Search strategy
Pubmed	(“health workforce”[tiab] OR “health personnel”[tiab] OR “health care worker”[tiab] OR “health personnel”[tiab] OR “health manpower”[tiab] OR nursing[tiab] OR “health care providers”[tiab] OR “healthcare staff”[tiab] OR “health professionals”[tiab]) AND (retention[tiab] OR detention[tiab] OR reservation[tiab] OR detainment[tiab] OR durability[tiab] OR permanence[tiab] OR persistence[tiab] OR recruitment[tiab] OR “personnel turnovers”[tiab] OR “personnel turnover”[tiab] OR willingness[tiab]) AND (disaster[tiab] OR pandemic[tiab] OR epidemic[tiab] OR “biological event”[tiab] OR “nuclear disaster”[tiab] OR “biological disaster”[tiab] OR “natural disaster”[tiab])
Embase	(“Personnel Turnover”:ti.ab.kw OR “Personnel Turnovers”: ti.ab.kw OR “Employee Turnover”:ti.ab.kw OR “Employee Turnovers”:ti.ab.kw OR “willingness to work”:ti.ab.kw OR “willingness to response”:ti.ab.kw) AND (“health workforce”:ti.ab.kw OR “Health Occupations Manpower”:ti.ab.kw OR “Health Manpower”:ti.ab.kw OR “personnel”:ti.ab.kw OR “clinical staff”) AND (“disasters” OR “disaster planning” OR “emergency” OR “pandemic”:ti.ab.kw OR “epidemic”:ti.ab.kw OR “biological event”:ti.ab.kw OR “nuclear event”:ti.ab.kw)
Web of Science	(TS = (“Personnel Turnover”) OR TS = (“ Personnel Turnovers”) OR TS = (“Employee Turnover”) OR TS = (“Employee Turnovers”) OR TS = (“willingness to work”) OR TS = (“willingness to response”)) AND (TS = (“health workforce”) OR TS = (“Health Occupations Manpower”) OR TS = (“Health Manpower”) OR TS = (personnel) OR TS = (“clinical staff”)) AND (TS = (“disaster planning”) OR TS = (emergency) OR TS = (pandemic) OR TS = (epidemic) OR TS = (“biological event”) OR TS = (“nuclear event”))
Scopus	(TITLE-ABS-KEY(“Personnel Turnover”) OR TITLE-ABS-KEY(“Personnel Turnovers”) OR TITLE-ABS-KEY(“Employee Turnover”) OR TITLE-ABS-KEY(“Employee Turnovers”) OR TITLE-ABS-KEY(“willingness to work”) OR TITLE-ABS-KEY(“willingness to response”)) AND (TITLE-ABS-KEY(“health workforce”) OR TITLE-ABS-KEY(“Health Occupations Manpower”) OR TITLE-ABS-KEY(“Health Manpower”) OR TITLE-ABS-KEY(personnel) OR TITLE-ABS-KEY(“clinical staff”)) AND (TITLE-ABS-KEY(“disaster planning”) OR TITLE-ABS-KEY(emergency) OR TITLE-ABS-KEY(pandemic) OR TITLE-ABS-KEY(epidemic) OR TITLE-ABS-KEY(“biological event”) OR TITLE-ABS-KEY(“nuclear event”))

### Inclusion and exclusion criteria

#### Subject

We included all the articles that consisted of the following: (1) review of challenges and factors affecting the maintenance of manpower; (2) manpower active in the health system, and (3) emergency due to natural disasters and the spread of new diseases. Articles that examined similar cases in organizations other than health were excluded.

#### Participants

All articles that contained the challenges of maintaining health manpower or the factors affecting them in emergencies were included in the study. Studies examining unrelated human resources were omitted.

### Design of the study

All case studies and reviews and articles that reported the successful experience of countries concerning maintaining health personnel in various crises and disasters were included in the study. Letters, abstracts of papers presented at conferences and seminars were also excluded from the study.

### Language

All articles published in English were included in the study.

### Study selection process

All articles obtained from the databases were screened by two researchers at different stages. In the screening stages after the elimination of duplicate studies, the titles of all articles were reviewed and articles that were not consistent with the objectives of the study were excluded from the study. In the next steps, the abstract and full text of the articles were studied, respectively, and studies that did not include inclusion criteria and had a weak relationship with the objectives of the study were identified and discarded. To ensure the richness of the articles, the final summary was reviewed and approved by human resources experts in health, emergency and disaster. Endnote X9 resource management software was used to organize resources.

### Extracting the data

In order to extract the data required for the study, first, the data of five articles were extracted experimentally using a researcher-made form that was manually designed by researchers in the Microsoft Word 2016 software environment, and the shortcomings and problems in the initial form were eliminated. The whole systematic review process was done by two authors and the disputes between the two were referred to a third party.

### Data analysis

After extracting the information by the data extraction form, the extracted information was analyzed using framework analysis. Framework analysis is a hierarchical approach used to classify and organize data based on key themes, concepts, and emerging classes. Recently, the framework analysis method has become popular as one of the tools for analyzing qualitative data obtained from health research, because it can be used for data management and systematic analysis. This approach allows the researcher to perform while examining the data in-depth, accurate and effective audits which enhances the accuracy of the data analysis and validation processes, simultaneously [8, 9].

The framework analysis approach has many similarities to thematic analysis, especially in the early stages, when repetitive and meaningful topics are identified. Therefore, analytical frameworks are like thematic networks. Also, the framework approach places great emphasis on data analysis and the relationships between (different) stages of analysis. The benefit of framework analysis is the development of a schematic and systematic framework for the analysis process so that research sponsors and others can understand the steps by which results are extracted from the data collected.

Framework analysis has seven main stages (transcription, interview familiarity, coding, creating an analytical work framework, analytical framework, charting data in the framework matrix, and interpreting the data). Although in frame analysis, data can be collected and analyzed simultaneously, the analysis can be performed in the form of these seven steps and in a linear process in which data are collected before the analysis begins [9].

## Results

**Fig. 1:**
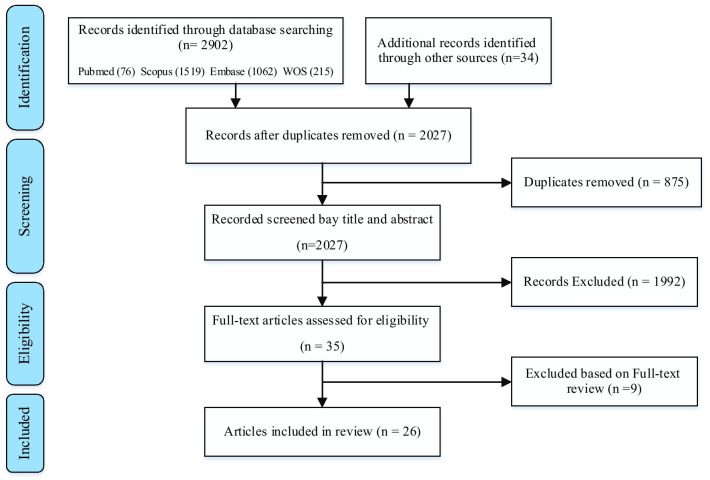
Process for identifying literature on willingness to work under disaster condition

Out of 2902 articles obtained from databases and searching of other sources, 875 articles were omitted due to being in common. In the screening process based on titles and abstracts, 1992 articles were removed. In reviewing the full text of the articles, nine cases were excluded from the study due to insufficient and irrelevant information. Finally, 26 articles were included in the study according to the inclusion and exclusion criteria (Fig. 1). All information related to the purpose of the study is reported in the extraction table (Additional file 1).

### Results according to countries

**Fig. 2:**
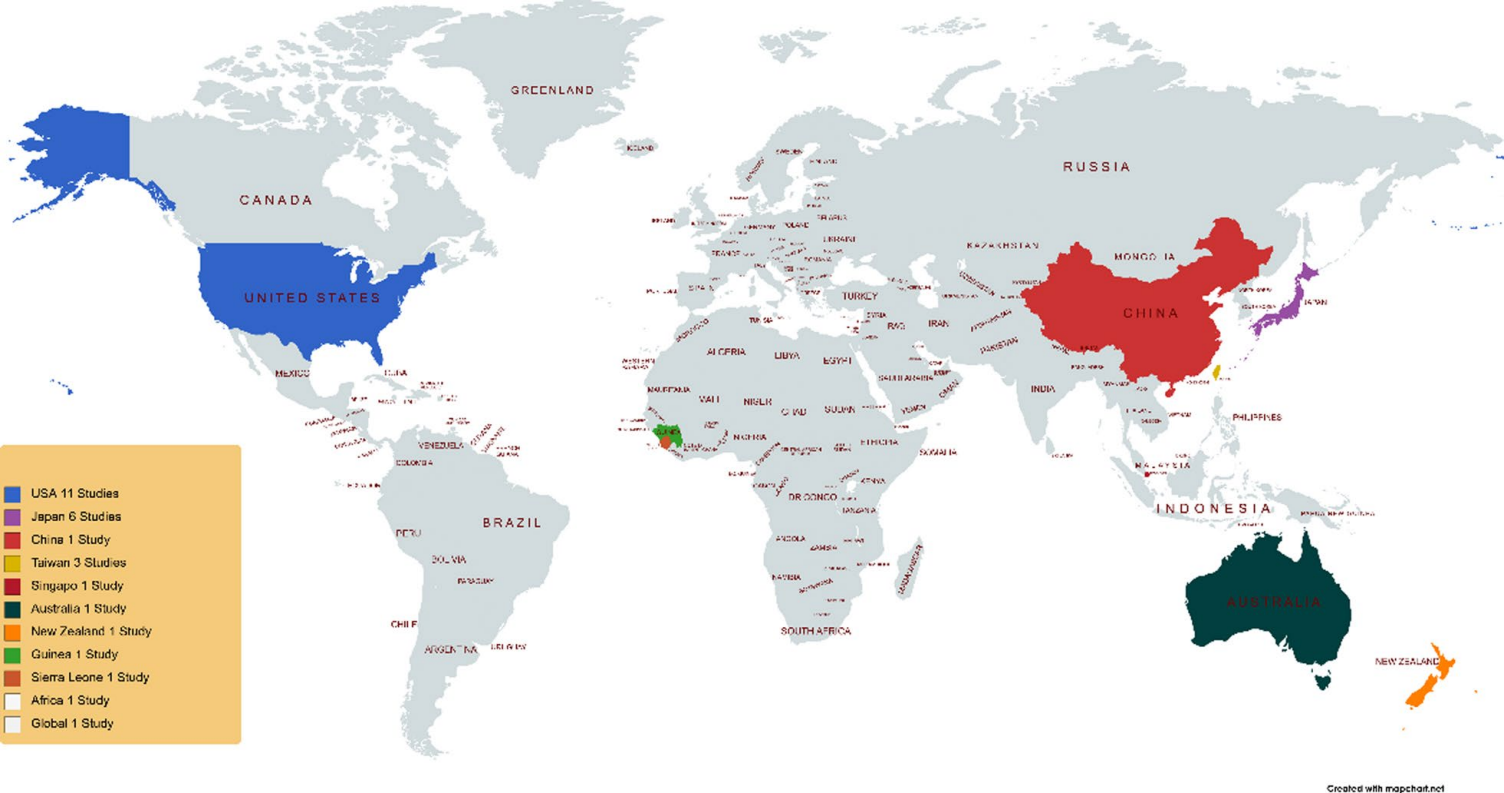
Geographical distribution of studies

The results of the present study show that out of 26 studies, 24 studies were conducted in nine countries (USA, Japan, China, Taiwan, Guinea, Sierra Leone, Australia, New Zealand and Singapore) and only 2 articles were conducted with the participation of several countries. The highest number of studies was done in the United States with 11 studies. Most of the countries that paid the most attention to this issue and the studies were conducted in were those countries that have high income and stable economies. Figure 2 illustrates the location of the included studies.

The results of the study indicate differences in interventions and strategies based on the level of income of countries, for example in the United States, Japan, Singapore, Australia, and New Zealand, which are known as high-income, the tendency to retain health professionals rely on education and training, creating a positive work atmosphere, personal safety, and family supportive whereas in China, which is classified as upper and middle-income, the most important factor that can sustain the health workers is psychological support of health personnel. In low-income countries such as African countries, especially New Guinea and Sierra Leone, the Ebola epidemic has shown that the biggest problem is in the health system and related policies, and if appropriate reforms are made, especially human resource management can make significant progress in the sustainability of health sector forces.

### Results based on the setting of the included studies

**Fig. 3:**
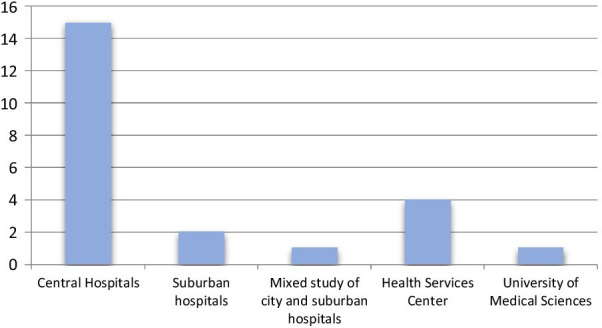
The setting of the studies

In the studies, 15 studies were conducted in the central hospitals, 2 studies in the suburban hospitals, 1 mixed study in the city and suburban hospitals, 4 studies in health and medical service centers, and 1 study in the University of Medical Sciences (Fig. 3).

### The method of included studies

**Fig. 4:**
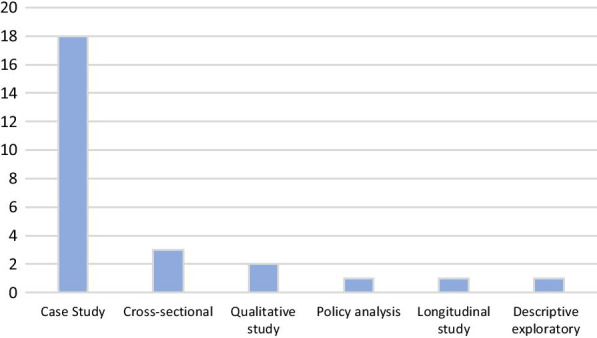
The design of studies

According to the study method of the articles included in the study, it was found that the highest number of articles was done by the case study method (18 articles), and the lowest number of articles was done by policy analysis, longitudinal study, and descriptive exploratory methods (Fig. 4).

### Disaster type of included studies

**Fig. 5:**
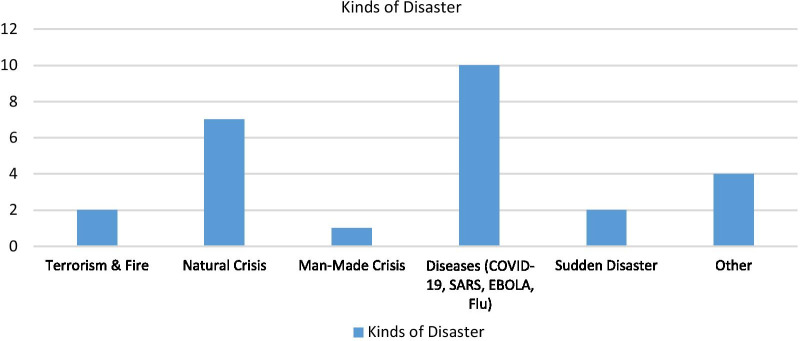
Kinds of disaster

The results of reviewing the studies showed that most types of crises are related to emerging and re-emerging diseases (10 studies). Earthquakes were also the most common natural disaster. Studies related to the man-made disaster were the lowest (Fig. 5).

### Barriers and challenge classification policy

After extracting data from studies in the two areas of barriers and solutions, due to the global nature of the study, with expert consultation, it was decided to classify them by a common international language based on the framework of the World Health Organization 6BB (Six building blocks). Studies have shown that the WHO framework is a strengthening tool for the health system and its use as an accelerator to achieve development goals (SDGs) is resilient. This framework, which includes providing service, human resources, information, medical products/vaccines and technology, finance and leadership, incorporates most of the findings obtained from the study to strengthen the resilience of human resources in crises and disasters and to create a resilient system. In this context, providing appropriate service is effective, safe, combined with qualified health interventions at the required time and place with minimal waste of resources, for the individual, the community, and those in need to strengthen, and with the retention of manpower in disaster, this goal can be achieved.

### Barriers and challenges identified

**Fig. 6:**
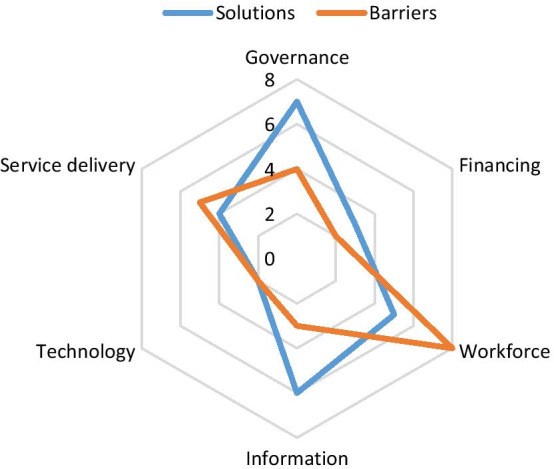
Barriers and solution identified

In general, in the section on the study of barriers and challenges in the retention of manpower 114 of the initial code were identified as the results of the studies. In the next step, by omitting duplicate codes and merging similar and homogeneous items, the number of codes was reduced to 24. The research team classified these identified codes into six main categories (24 subcategories) based on a 6bb framework. The highest recurrence of the challenge was in the human resources category, including poor leadership communication and emotional support, and family worries, and the lowest recurrence in the financial category, including lack of budget of training, salaries and compensation of personnel (Fig. 6).

### Results based on solutions and interventions

According to the results of the articles included in the study, 133 primary codes related to solutions and interventions for manpower maintenance in emergencies and disasters were identified by the research team. After removing the duplicate codes and merging the homogeneous items, the codes were reduced to 27 items. After categorizing the codes by the researchers, the extracted items were divided into six main categories (27 subcategories) according to the six building blocks framework category. Most of the solutions are related to the management class with seven subcategories, which are mostly in the field of positive communication with staff and outside the organization; the least repetition in the financial class with support programs and resource development for hiring and retention.

**Fig. 7:**
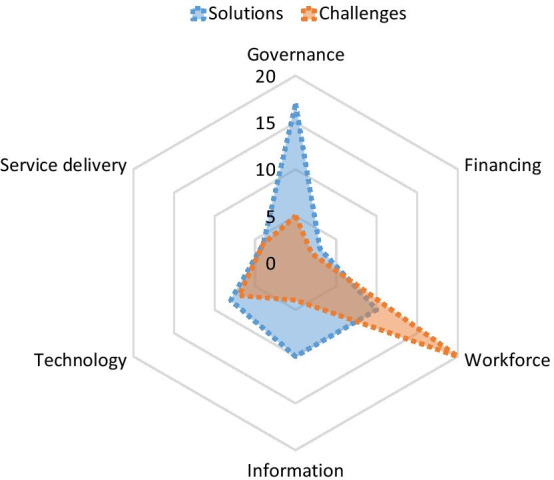
Solutions and interventions identified

The combination of barriers and solutions is presented in Fig. 7 schematically, showing that the problems were more in the field of human resources and the presentation of solutions is more in the discussion of management and leadership.

The highest challenges related to human resources retention consist of weak relationship with personnel, lack of emotional support, fear of infecting, moral dilemmas and patient care with fear, and lack of training budget and staff salaries and compensation. Table 2 indicates a list of essential considerations for health policy-makers after synthesis of results.

**Table 2:** Key considerations for health policy-makers that arose from the literature review

Consideration	Supporting literature
Rewards, safety, and high-level care make employees committed	[13]
Psychological care and improving the working conditions of healthcare providers play an important role in staff sustainability	[14, 15]
Ensure that personal protective equipment/vaccines and antiviral drugs are available to hospital staff Risk assessment and designing training programs to respond to emergency	[16, 17]
Counseling and providing supportive and psychiatric care to health care providers and their family members	[18]
Increase cooperation between governments and health service providers in attracting and retaining human resources for health	[19]
Providing welfare and support facilities to the children of health workers	[20, 21]
Effective leadership, providing the necessary resources in the shortest possible time	[22]
Empowering health workers, especially nurses, in the face of emergency condition (crisis maneuvers)	[23]
Existence of specific instructions for providing services in crisis situations Existence of a crisis plan in a hospital or medical center	[24]
Supporting the career development of nurses Increasing the authority and participation of nurses in critical situations	[25]
Existence of protocols for attracting and supporting volunteers and students	[26]

## Discussion

This research uses the systematic mapping review method to examine the challenges that medical staff face in the time of emergency and disaster and its aim is to provide solutions for this issue. In this study, 26 articles from 11 countries were identified and reviewed. The United States of America has published the largest number of articles (11 articles) in recent years and is one of the countries that paid the most attention to this field. Besides, the most important issues and crises that have been raised in these countries were the crises related to diseases.

The most important challenges that countries identified were as follows: poor relationship of managers with personnel and lack of emotional support, family concerns, job security after returning to work, personnel fatigue and stress, moral dilemmas, lack of following the health protocols, patient care concomitant with fear, lack of presence of senior managers in the front line, inadequate training and practices, personnel pay and compensation system, problems in digital and health substructures, and structural weaknesses of the hospitals. Also, based on studies, the solutions were: good communication of managers with personnel and organizations, developing supporting policies, enactment of maintenance regulations, providing and updating health instructions, developing the organizational culture and loyalty, public and religious support and appreciation, localization and using volunteers, enhancing the sense of responsibility and confidence, providing medicine and equipment, increasing the capacity of hospitals, and providing facilities for personnel and their families [27–30].

The results of this study show that most of the researches about the maintenance of human resources at the time of disaster are conducted in rich and semi-rich countries. Due to the incidence of epidemics like Ebola and its severe impact on the absence of health staff, low-income countries need to do further researches on the subject. One of the reasons that countries like the United States and Japan are more concerned about the maintenance of human resources at the time of disaster is the fact that these countries have a high occurrence of natural disasters, which consequently increases the need for the presence of human resources. The other reasons that reduce the presence of human resources in low-income countries can be financial problems and the risk of being exposed to contagious diseases [17, 19, 24, 31]. Besides, the results of the study show that for the reason of different prioritization between high-income and low-income countries, their solutions for protecting personnel at the time of disaster are different. To illustrate this, in high-income countries the corporeal and mental support of personnel at the time of disaster or emergency is considered the first priority but in low-income countries the financial issues are raised as priorities [14, 18, 32].

According to the results of this study, the shortage of workforce occurs at the time of natural and human-made disasters, epidemics, and pandemics. In the last 20 years, the shortage of workforce has more often occurred at the time of the outbreak of new and recurring diseases when comparing to natural and human-made disasters. It can be related to the reoccurrence of these types of crises and the associated health, family, and society concerns about them. These concerns will consequently raise the absenteeism rate of personnel. On the contrary, personnel’s challenges at the time of natural and human-made disasters are the security of their families and the security of the path between their home and workplace. In this regard, health systems must consider and solve these challenges before their occurrence so that they can benefit from the presence of committed and experienced personnel at the time of disaster [15, 16, 20, 32–34]. Moreover, countries can prepare themselves for such situations by enacting mandatory laws and regulations about the health systems. For instance, the United States has appointed a joint committee to appraise the condition of healthcare centers and also has increased the capacity of hospitals for the time of disaster.

The results of the study also show that most of the researches have been conducted in hospital environments which shows that hospitals are the main centers and the front line confronting disasters [13, 21, 23, 25, 26, 35, 36]. For this reason, they require special attention from officials to provide them sufficient human resources and equipment. Increasing the preparedness of hospitals is an inevitable measure to reduce their risks. The safety index of hospitals which is presented by the World Health Organization can also be helpful; because it evaluates the preparedness of hospitals according to its structural, non-structural, and practical criteria. Using technology to build and develop smart hospitals can also prevent wasting of resources and shortage of personnel and can be helpful to the healthcare system of countries.

The findings of the present study also show that one of the most important challenges that make it difficult to maintain human resources is the negligence of managers about the personnel’s emotional needs and the existence of a poor relationship between them. Separation from family, which consequently leads to mental fatigue, can put personnel in a dilemma and cause their absence [22, 37, 38]. One of the solutions that can be effective in such conditions is to hire adequate human resources and adjust their shifts appropriately to prevent staff burnout due to hard work and separation from family. By these measures, the required personnel are available at any time.

### Limitations

One of the limitations of this study was that only English documents and researches have been reviewed; however, other successful researches might be in different languages that have not been reviewed in this study. Another limitation of the study was the small number of researches about the maintenance of human resources during disasters and crises. Regarding the frequency of crises during the last 10 years, further studies about this field are required. Another issue was that most of the studies had only considered nurses while a wide range of staff work in health systems. Besides, the quality of studies, statistical heterogeneity, and the effect of different demographic factors may have influenced the results of this study.

The findings of the study show that most of the researches have been conducted in hospitals while the healthcare centers are actually the front line of prevention. In other words, healthcare centers have a great function in reducing the workload of hospitals, preventing the burnout and absence of personnel, and reducing the cost of complementary treatments. According to the findings of the study, universities can also educate and train personnel and prepare them for a professional confrontation with crises. Other key points are the managers’ support and the personnel’s confidence and readiness to face the disaster. A qualified manager tries to support his employees emotionally and mentally, while the lack of senior managers’ support was evident during the disaster. Further studies and training programs in this field can also be effective. Furthermore, the absence of personnel was specially increased at the time of new and recurring diseases. The reason can be related to personal and family health concerns. This problem can be solved by additional training about the infection control and personal care in the educational programs of universities and health service centers. Awareness will reduce fear and worries and can help the staff to attend their job.

Another practical solution is to provide smart and long-distance medical services, especially for geographical areas prone to crises and disasters regarding the fact that no one should be deprived of receiving medical services. This measure can protect personnel from being exposed to different risks and enables them to continue the service process even in the shortage of workforce.

Finally, regarding the number of researches, most researches were conducted in rich and semi-rich countries and are not applicable to other countries due to the different economic and political conditions. The low-income countries are recommended to conduct further studies due to their population, various crises they face, and weaknesses of their health system.

## Conclusion

This literature review has sought to map out the willingness to work in healthcare providers in emergency conditions. This article has tried to provide a comprehensive and detailed analysis of the issue for developing evidence-based policy and interventions. According to this investigation, policy-makers should improve disaster management and planning skills in healthcare managers and staffs. Besides, psychological and mental support, disaster education and holding maneuver and exercises are important measures to increase preparedness of health care workers and decrease absenteeism.

## Supplementary Information

**Additional file 1.** Table of all studies included in the review.

## Data Availability

The data that support the findings of this study are available from the corresponding author upon reasonable request.

## References

[1] Fischbacher-Smith D, Fischbacher-Smith M (2013). Tales of the unexpected: issues around the development of a crisis management module for the MBA program. J Manag Educ.

[2] Shafii M, Hashemi FS, Askari R, Pakdaman M, Bahariniya S. Estimation of the Required Staffing Capacity of Selected Hospitals in Yazd City, Iran, in Accordance with Staffing Standards of Iranian Ministry of Health and Medical Education in Year 2017. 2019.

[3] Rousseaux F, Lhoste K, editors. Process analysis, modeling and simulation for crisis management. 2008 International Workshop on Advanced Information Systems for Enterprises; 2008: IEEE.

[4] Liu Y, Guo Y, editors. Organizational Crisis Management and Public Policy Problem. 2012 Second International Conference on Business Computing and Global Informatization; 2012: IEEE.

[5] Loizou M, Hartley T, Slater S, Newman R, Pannese L, editors. Emotions for intelligent agents in crisis management simulations: a survey. 2012 17th International Conference on Computer Games (CGAMES); 2012: IEEE.

[6] Clapton J, Rutter D, Sharif N (2009). SCIE systematic mapping guidance.

[7] Qin X, Jiang Y (2011). The impact of natural disaster on absenteeism, job satisfaction, and job performance of survival employees: an empirical study of the survivors in Wenchuan earthquake. Front Bus Res China.

[8] Blaxter M (1996). Criteria for the evaluation of qualitative research papers. Med Sociol News.

[9] Srivastava A, Thomson SB. Framework analysis: a qualitative methodology for applied policy research. 2009.

[10] Williams J, Nocera M, Casteel C (2008). The effectiveness of disaster training for health care workers: a systematic review. Ann Emerg Med.

[11] Gale NK, Heath G, Cameron E, Rashid S, Redwood S (2013). Using the framework method for the analysis of qualitative data in multi-disciplinary health research. BMC Med Res Methodol.

[12] Cowden J, Crane L, Lezotte D, Glover J, Nyquist AC (2010). Pre-pandemic planning survey of healthcare workers at a tertiary care children’s hospital: ethical and workforce issues. Influenza Other Respir Viruses.

[13] Wagner CM, Huber DL (2003). Catastrophe and nursing turnover: nonlinear models. Jona J Nursing Adm..

[14] Fujitani K, Carroll M, Yanagisawa R, Katz C (2016). Burnout and psychiatric distress in local caregivers two years after the 2011 Great East Japan Earthquake and Fukushima nuclear radiation disaster. Community Ment Health J.

[15] Chersich MF, Gray G, Fairlie L, Eichbaum Q, Mayhew S, Allwood B (2020). COVID-19 in Africa: care and protection for frontline healthcare workers. Glob Health.

[16] Balicer RD, Barnett DJ, Thompson CB, Hsu EB, Catlett CL, Watson CM (2010). Characterizing hospital workers’ willingness to report to duty in an influenza pandemic through threat-and efficacy-based assessment. BMC Public Health.

[17] Mohamed K, Rodríguez-Román E, Rahmani F, Zhang H, Ivanovska M, Makka SA (2020). International efforts to save healthcare personnel during COVID-19. Acta Bio Medica: Atenei Parmensis..

[18] Kang L, Ma S, Chen M, Yang J, Wang Y, Li R, et al. Impact on mental health and perceptions of psychological care among medical and nursing staff in Wuhan during the 2019 novel coronavirus disease outbreak: a cross-sectional study. Brain, Behav, Immunity. 2020. 10.1016/j.bbi.2020.03.028 PMC7118532 32240764

[19] van de Pas R, Kolie D, Delamou A, Van Damme W (2019). Health workforce development and retention in Guinea: a policy analysis post-Ebola. Hum Resour Health.

[20] Hirohara M, Ozaki A, Tsubokura M (2019). Determinants and supporting factors for rebuilding nursing workforce in a post-disaster setting. BMC Health Serv Res.

[21] Adams LM, Berry D. Who will show up? Estimating ability and willingness of essential hospital personnel to report to work in response to a disaster. Online J Issues Nursing. 2012;17(2). 22686116

[22] Stanz L, Weber RJ. Leadership approaches to staff health and wellness during COVID-19 pandemic. Hosp Pharm. 2020:0018578720936589. 10.1177/0018578720936589 PMC8559045 34732914

[23] Baack S, Alfred D (2013). Nurses’ preparedness and perceived competence in managing disasters. J Nurs Scholarsh.

[24] Mitani S, Kuboyama K, Shirakawa T (2003). Nursing in sudden-onset disasters: factors and information that affect participation. Prehosp Disaster Med.

[25] Nakayama Y, Kato I, Ohkawa T (2019). Sustaining power of nurses in a damaged hospital during the Great East Japan earthquake. J Nurs Scholarsh.

[26] Yonge O, Rosychuk RJ, Bailey TM, Lake R, Marrie TJ (2010). Willingness of university nursing students to volunteer during a pandemic. Public Health Nurs.

[27] Barnett DJ, Thompson CB, Errett NA, Semon NL, Anderson MK, Ferrell JL (2012). Determinants of emergency response willingness in the local public health workforce by jurisdictional and scenario patterns: a cross-sectional survey. BMC Public Health.

[28] Chang CS, Du PL, Huang IC (2006). Nurses’ perceptions of severe acute respiratory syndrome: relationship between commitment and intention to leave nursing. J Adv Nurs.

[29] Davidson JE, Sekayan A, Agan D, Good L, Shaw D, Smilde R (2009). Disaster dilemma: factors affecting decision to come to work during a natural disaster. Adv Emerg Nurs J.

[30] Walsh L, Craddock H, Gulley K, Strauss-Riggs K, Schor KW (2015). Building health care system capacity: training health care professionals in disaster preparedness health care coalitions. Prehosp Disaster Med.

[31] Raven J, Wurie H, Witter S (2018). Health workers’ experiences of coping with the Ebola epidemic in Sierra Leone’s health system: a qualitative study. BMC Health Serv Res.

[32] Qureshi K, Gershon RR, Sherman MF, Straub T, Gebbie E, McCollum M (2005). Health care workers’ ability and willingness to report to duty during catastrophic disasters. J Urban Health.

[33] Shiao JS-C, Koh D, Lo L-H, Lim M-K, Guo YL (2007). Factors predicting nurses’ consideration of leaving their job during the SARS outbreak. Nurs Ethics.

[34] Imai H, Matsuishi K, Ito A, Mouri K, Kitamura N, Akimoto K (2010). Factors associated with motivation and hesitation to work among health professionals during a public crisis: a cross sectional study of hospital workers in Japan during the pandemic (H1N1) 2009. BMC Public Health.

[35] Scrymgeour G, Smith L, Maxwell H, Paton D (2020). Nurses working in healthcare facilities during natural disasters: a qualitative enquiry. Int Nurs Rev.

[36] Morioka N, Tomio J, Seto T, Kobayashi Y (2015). Trends in the geographic distribution of nursing staff before and after the Great East Japan Earthquake: a longitudinal study. Hum Resour Health.

[37] Rangachari P, Woods JL (2020). Preserving organizational resilience, patient safety, and staff retention during COVID-19 requires a holistic consideration of the psychological safety of healthcare workers. Int J Environ Res Public Health.

[38] Koh D, Lim MK, Chia SE, Ko SM, Qian F, Ng V, et al. Risk Perception and impact of severe acute respiratory syndrome (SARS) on work and personal lives of healthcare workers in singapore what can we learn? Med Care. 2005:676–82. 10.1097/01.mlr.0000167181.36730.cc 15970782

